# Animal Exposure Model for Mapping Crimean-Congo Hemorrhagic Fever Virus Emergence Risk

**DOI:** 10.3201/eid3004.221604

**Published:** 2024-04

**Authors:** Sara Baz-Flores, Débora Jiménez-Martín, Alfonso Peralbo-Moreno, Cesar Herraiz, David Cano-Terriza, Raúl Cuadrado-Matías, Ignacio García-Bocanegra, Francisco Ruiz-Fons

**Affiliations:** Instituto de Investigación en Recursos Cinegéticos, Ciudad Real, Spain (S. Baz-Flores, A. Peralbo-Moreno, C. Herraiz, R. Cuadrado-Matías, F. Ruiz-Fons);; Universidad de Córdoba, Córdoba, Spain (D. Jiménez-Martín, D. Cano-Terriza, I. García-Bocanegra);; Instituto de Salud Carlos III, Madrid, Spain (D. Cano-Terriza, I. García-Bocanegra, F. Ruiz-Fons)

**Keywords:** Crimean-Congo hemorrhagic fever, CCHF, epidemiology, Hyalomma, orthonairovirus, serologic survey, small ruminants, ticks, viruses, parasites, vector-borne infections, zoonoses, Spain

## Abstract

To estimate the determinants of spatial variation in Crimean-Congo hemorrhagic fever virus (CCHFV) transmission and to create a risk map as a preventive public health tool, we designed a survey of small domestic ruminants in Andalusia, Spain. To assess CCHFV exposure spatial distribution, we analyzed serum from 2,440 sheep and goats by using a double-antigen ELISA and modeled exposure probability with environmental predictors by using generalized linear mixed models. CCHFV antibodies detected in 84 samples confirmed low CCHFV prevalence in small domestic ruminants in the region. The best-fitted statistical model indicated that the most significant predictors of virus exposure risk were cattle/horse density and the normalized difference vegetation index. Model validation showed 99.7% specificity and 10.2% sensitivity for identifying CCHFV circulation areas. To map CCHFV exposure risk, we projected the model at a 1 × 1-km spatial resolution. Our study provides insight into CCHFV ecology that is useful for preventing virus transmission.

Crimean-Congo hemorrhagic fever (CCHF) is a tickborne zoonosis caused by CCHF virus (CCHFV). The World Health Organization considers CCHF one of the highest priority diseases because of its epidemic potential, its high case-fatality rate (10%–40%), and its difficult prevention and treatment (*1*). Clinical disease is restricted mainly to humans, but the virus can infect a wide range of animal species (*2*). CCHFV infections in animals are mainly asymptomatic, which complicates detection of the virus and increases the risk for human infection. Humans can become infected by the bite of a CCHFV-infected tick or through direct contact with virus-contaminated tissues or blood (*3*). Although some outbreaks are associated with high case-fatality rates, most (≈90%) human infections are asymptomatic or cause mild illness (*2*). Cases of CCHF are associated with rural areas. Veterinarians, farmers, hunters, environmental rangers, and abattoir personnel are at highest risk for infection (*4*).

CCHFV is prevalent in Africa, eastern Europe, the Middle East, and across central Asia to western China (*5*). In the 21st century, the geographic range and incidence of confirmed CCHF cases have markedly increased (*2*). Climate change and landscape transformations have affected the abundance and spatial range of CCHFV animal hosts and vectors (*6*), strongly influencing CCHFV transmission dynamics (*7*) and modifying the likelihood of disease emergence and re-emergence (*4*). Those changes are the most likely underlying reason for the emergence of CCHF in Spain.

Exposure to CCHFV on the Iberian Peninsula (mainland Spain and Portugal) was first evidenced in humans in Portugal in 1985 (*8*), but the first confirmed clinical case was reported in 2016 in Spain (*9*). Since then, 12 human cases (4 deaths) have been reported in Spain (*10*,*11*). Because no vaccine is available, humans in or near CCHFV-endemic areas are advised to take precautions when spending time in nature (or tick-prone areas), including limiting skin exposure, applying tick repellents, and thoroughly inspecting the skin after field activities. Identifying spatiotemporal virus transmission hotspots may provide information for surveillance and prevention strategies to reduce exposure to CCHFV. Although the likelihood of virus exposure within the general population is low (*12*) because of a predominantly urban lifestyle, greater accuracy in risk prediction may lead to more effective preventive measures for the at-risk population (*13*).

CCHFV has been detected in several species of ticks, but the major CCHFV reservoirs and vectors are considered to be *Hyalomma* spp. ticks (*14*). Two species of *Hyalomma* ticks transmit CCHFV in the Iberian Peninsula, *H. lusitanicum* and *H. marginatum*, and both are abundant in southwestern Spain (*15*–*17*). In general, CCHFV circulates in a silent enzootic tick-vertebrate-tick cycle, the balance of which relies on a complex animal-tick-environment interplay. However, horizontal transmission (cofeeding, transstadial) and vertical transmission (transovarial) can occur within the tick population (*18*). In vertebrate animals, excluding humans, only a transient viremia (≈5 days) develops after infection, but those animals are essential hosts to *H. lusitanicum* and *H. marginatum* ticks and thus play a fundamental role in the spread of CCHFV.

Seroepidemiologic studies in animals can be useful for localizing CCHFV foci and providing information for future research efforts and prevention actions. Farm animals closely coexist with humans and have been epidemiologically linked to human CCHF cases. Therefore, those animals could be used for surveillance purposes (*19*–*21*). Small domestic ruminants (sheep and goats) are abundant in Spain. Indeed, Spain hosts the largest sheep population and the second largest goat population in the European Union (*22*). Direct or indirect interactions between those animals and wild ungulates (e.g., red deer [*Cervus elaphus*] or Eurasian wild boar [*Sus scrofa*]) may be frequent, and both species play major roles in maintaining tick populations (*16*,*17*). Thus, *H. lusitanicum* and *H. marginatum* ticks are abundant on domestic ruminants (*23*).

Because seroepidemiologic studies in animals, along with identification of CCHFV ecologic drivers, can provide insights into CCHFV transmission dynamics (*13*), resulting in better preventive strategies for the human population at risk, we designed a cross-sectional serosurvey of domestic small ruminants in a CCHFV-enzootic region of Spain, Andalusia (*17*,*24*), and statistically modeled exposure risk with environment-associated predictors to map infection risk hotspots. Our working hypothesis was that estimating ecologic drivers of CCHFV exposure risk in small domestic ruminants would reveal the spatial risk for virus transmission to humans. That information would help with the design of ad hoc public health preventive actions in CCHFV-enzootic regions (*13*,*25*,*26*).

The collection of blood samples analyzed was part of the official Animal Health Campaigns of Regional Government of Andalusia, Spain. Therefore, no ethics approval was necessary.

## Materials and Methods

### Study Design

**Figure 1:**
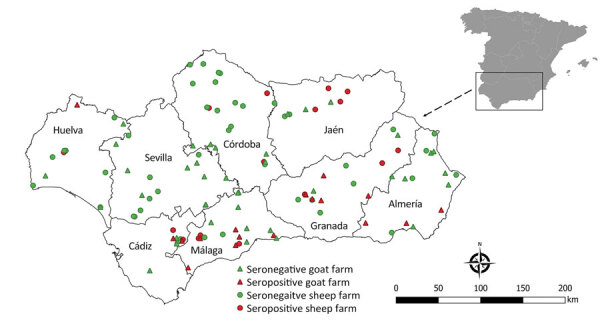
Location of farms in study of animal exposure model for mapping Crimean-Congo hemorrhagic fever virus emergence risk, Andalusia, Spain. Inset shows location of Andalusia within mainland Spain.

To analyze the prevalence of antibodies against CCHFV in randomly selected small ruminant farms at both the animal and herd levels, during December 2015–February 2017, we conducted a cross-sectional serosurvey in Andalusia (southern Spain: 36°N–38°60′N, 1°75′W–7°25′W; Figure 1). Andalusia is the first stopover in Europe for birds annually migrating from Africa to western Europe that may carry CCHFV-infected *Hyalomma* spp. ticks. We know that CCHFV circulates enzootically in large areas of Andalusia (*13*,*25*,*26*), but we do not know the actual distribution of the virus in the region.

We randomly selected 122 farms (61 sheep farms and 61 goat farms) according to the stratified census of small ruminant farms per province. Further details on farm selection criteria have been published (*27*). We estimated the minimum number of samples per farm required to estimate antibody prevalence at the previously known circulation rates in southwestern Europe (5%) (*28*) to be 20 with a 95% CI level and an accepted 10% error by using the sample size to estimate a proportion with specified precision calculator of Epitools (https://epitools.ausvet.com.au). Subsequently, within each farm, we randomly sampled 20 small ruminants. Blood samples were obtained by jugular vein puncture and transported to the laboratory and centrifuged at 400 × *g* for 10 minutes to obtain serum that was stored at −20°C until analysis.

### Serologic Analyses and Prevalence Calculations

We determined the presence of CCHFV antibodies by using a highly sensitive and specific commercial CCHF double-antigen multispecies ELISA kit (IDScreen CCHF Double Antigen Multispecies, https://www.innovative-diagnostics.com) according to the manufacturer’s instructions (*29*). We estimated the overall prevalence of antibodies from the ratio of positive samples to the total number of analyzed samples. We estimated the Clopper-Pearson exact 95% CI for any prevalence value obtained.

### Environmental Risk Factors

We performed statistical modeling with predictors selected from environmental factors (Table 1) that characterized the vicinity of the farm. We defined a 5-km radius buffer around farm location coordinates. We selected that buffer size on the basis of the maximum expected movement distance of extensive or semiextensive herds for consumption of local resources (temporary pastures, crop stubble, natural resources). The buffer size was selected to also account for any possible indirect influence of neighboring farms or surrounding wildlife on the risk for CCHFV exposure of domestic small ruminant herds. Only a small fraction of the farms (4/122 [3.3%]) reported seasonal long-distance movements that were thus considered irrelevant for determining the buffer size. The estimated predictor values were rescaled to the spatial scale of the selected buffer by weighted averaging.

**Table 1:** Set of explanatory predictors gathered for risk factor analyses used for mapping Crimean-Congo hemorrhagic fever virus emergence risk from animal exposure model

Factor, predictor	Description, unit of measure	Average (range)
Host-related		
** boveq**	**Cattle and horse summed density, ind/ha**	**0.08 (0–0.45)**
peqrum	Small ruminant density, ind/ha	0.51 (0.02–1.96)
rd	Red deer density, harvested ind/ha	0.04 (0–0.48)
wb	Eurasian wild boar density, harvested ind/ha	0.05 (0–0.24)
** otung**	**Other wild ungulate density, harvested ind/ha**	**0.13 (0–0.45)**
Bioclimatic		
lstinv	Mean winter land surface temperature, °C	15.77 (11.92–20.76)
lstver	Mean summer land surface temperature, °C	39.61 (32.36–45.12)
lstanu	Mean annual land surface temperature, °C	29.34 (23.01–33.60)
lstvarinv	Winter land surface temperature variation, °C	25.03 (12.32–45.47)
lstvarver	Summer land surface temperature variation, °C	21.06 (9.80–30.06)
lstvaranu	Annual land surface temperature variation, °C	121.77 (64.69–185.28)
NDVIinv	Winter normalized difference vegetation index	4,563.39 (1,969.59–7,002.13)
NDVIver	Summer normalized difference vegetation index	3,053.56 (1,235.00–5,665.86)
** NDVIanu**	**Annual normalized difference vegetation index**	**3915.94 (1708.02–6546.01)**
** NDVIvarinv**	**Winter normalized difference vegetation index variation**	**473,323.50 (95,507.99–2,331,259.00)**
NDVIvarver	Summer normalized difference vegetation index variation	126,977.60 (22,445.69–1,028,808.00)
** NDVIvaranu**	**Variance of the annual normalized difference vegetation index**	**1,051,955.00 (207,897.60–3,747,242.00)**
Land use-related		
matdi	Proportion of sparse shrubland in the buffer, %	0.11 (0–0.55)
matde	Proportion of dense shrubland in the buffer, %	0.08 (0–0.43)
** bos**	**Proportion of woodland in the buffer, %**	**0.06 (0–0.41)**

*Boldface indicates variables selected for modeling after the descriptive analysis. ind/ha, individuals per hectare.

#### Host-Related Predictors

Wild and domestic ungulate abundance is a relevant parameter in CCHF epidemiology (*13*,*17*). We gathered domestic ruminant census data from the 2009 national census (https://www.ine.es) on a regional veterinary unit level spatial scale. We used census data for cattle, horses, and small domestic ruminants to estimate 2 predictors: small domestic ruminant density and cattle/horse density. We used hunting bag data at hunting ground level from the 2014–15 through 2020–21 hunting seasons (kindly provided by the Andalusia regional government) as a proxy of wild ungulate relative abundance (*30*). We estimated 3 predictors: relative abundance of red deer, relative abundance of wild boar, and relative abundance of other wild ungulates.

#### Bioclimatic Predictors

 We selected 2 bioclimatic predictors from telemetry data—the land surface temperature (LST) and the normalized difference vegetation index (NDVI)—because of their potential effects on local tick abundance (*17*,*31*). Both parameters were obtained at a 1 × 1–km spatial resolution and at daily (LST) or 2-week (NDVI) temporal resolution for 2014–2016 from the MODIS website (https://modis.gsfc.nasa.gov). The NDVI is an indicator of plant photosynthetic activity that is associated with water availability and thus indicates the hydric stress that off-host ticks can experience. We estimated period average NDVI and LST and their variance for winter, summer, and the whole year. We estimated the average and variance for all 3 winters, 3 summers, and 3 years of the study and not for each year because we wanted to characterize each area, not compare between years. We selected winter and summer as the critical periods for tick survival and annual LST and NDVI as determinants of *Hyalomma* spp. tick activity (*32*).

#### Land Use–Related Predictors

As habitat predictors, we considered 3 land cover variables as favorable habitats for *Hyalomma* spp. (*17*,*32*) and wild ungulates (*33*): woodland, dense shrubland, and sparse shrubland cover. We obtained land-use data from the SIPNA database (https://portalrediam.cica.es/descargas). We estimated the proportion of each land cover type at the farm buffer selected scale.

### Risk Analyses and Mapping

To reduce the variability in measure scales of the continuous predictors, we applied a standardization rescaling process by using the scale function of R statistical software (https://cran.r-project.org). We explored relationships among continuous predictors by building a correlation matrix (chart.correlation function of the PerformanceAnalytics R package). Thereafter, we analyzed Y~X relationships and excluded specific predictors from the set of highly correlated variables (r>|0.7|) that had the lowest power to explain the variance of the response variable (*34*). After we finished the exploratory analysis, we analyzed the association of the selected predictors with the individual risk for exposure to CCHFV (antibody positive/negative, n = 2,400) by using generalized linear mixed-effects models (*35*) with the farm as random effect factor. We built and ranked all possible models by increasing corrected Akaike Information Criterion and using the dredge function of the R MuMIn statistical package. We selected the model with the lowest Akaike Information Criterion as the best-fit model (*36*) and validated its predictive potential by means of repeated k-fold cross-validation. We divided data into 10 groups (κ = 10) and repeated the cross-validation 50 times by using the cross-validate function of the cvms R package (*37*). Subsequently, we estimated average cross-validation values to get an overall predictive capacity of the model. After we validated the model, we projected it at a 1 × 1–km spatial resolution to map predicted risk for Andalusia. For that model, we estimated selected variables for the study period at the projection spatial scale for Andalusia. We considered the first farm of the series as the reference for projection. Model projection was performed by using the predict function of the car package in the R environment.

## Results

We detected antibodies against CCHFV in 84 of the 2,440 (3.4%, 95% CI 2.8%–4.2%) small ruminants tested. Exposure prevalence among sheep and goats was similar: sheep 3.0% (95% CI 2.1%–4.1%; 36/1,220) and goats 3.9% (95% CI 3.0%–5.2; 48/1,220). At least 1 seropositive animal was found in 16 of 61 (26.2%, 95% CI 16.8%–38.4%) surveyed sheep farms and in 18 of 61 (29.5%, 95% CI 19.6%-41.9%) goat farms. Overall, the number of farms with >1 seropositive animal was 34 of 122 (27.8%, 95% CI 20.7%–36.4%) (Figure 1).

**Figure 2:**
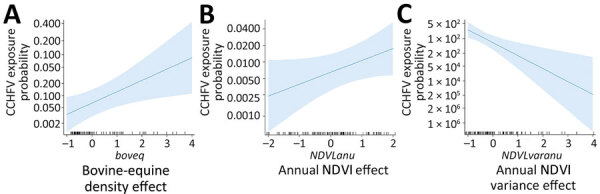
Model output charts displaying the effect of the best-fitted model selected variables on the risk of small ruminant exposure to Crimean-Congo hemorrhagic fever virus, Andalusia, Spain. Shaded areas indicate 95% CIs. NDVI, normalized difference vegetation index.

**Figure 3:**
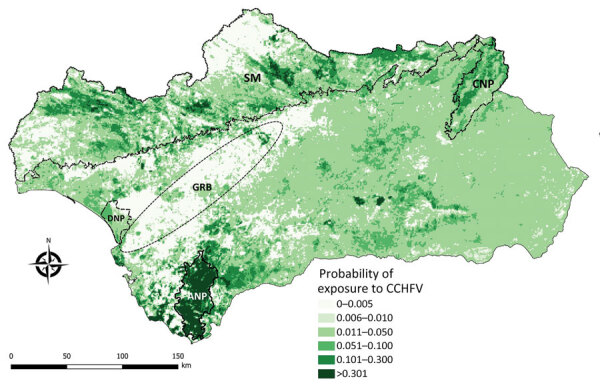
Spatial projection of model for risk for exposure of small ruminants to Crimean-Congo hemorrhagic fever virus in Andalusia, Spain. The model was projected at a 1 × 1–km spatial resolution. ANP, Los Alcornocales Natural Park; CNP, Sierras de Cazorla, Segura y Las Villas Natural Park; DNP, Doñana National Park; GRB, Guadalquivir River basin; SM, Sierra Morena mountain chain.

We excluded 2 farms (1 sheep farm and 1 goat farm) and a total of 40 (seronegative) animals from the risk factor analysis because of incorrect recording of location coordinates. The best-fitted model selected 3 of the considered predictors, including cattle/horse density, annual NDVI, and annual NDVI variance (Table 2). Cattle/horse density around small ruminant farms was significantly associated with exposure probability (Figure 2). A positive, albeit not statistically significant, relationship was also observed for the NDVI. In contrast, we observed a strong and statistically significant negative relationship between the annual NDVI variance and the risk for exposure of individual small domestic ruminants to CCHFV. The cross-validation analysis showed that the balanced accuracy of the model was 0.549, sensitivity was 10.2%, and specificity was 99.7%. Also, the model had good discriminatory power (area under the curve = 0.830). For a prior probability of infection of 0.05, the positive predictive value was 0.6415 and the negative predictive value was 0.9547. The spatial projection of the model showed lower predicted risk areas in central and eastern Andalusia and higher predicted risk areas north and south of the region (Figure 3).

**Table 2:** Output of the generalized linear mixed-effects model used to analyze the risk for exposure to Crimean-Congo hemorrhagic fever virus*

Predictor (see Table 1)	Estimate	SE	z	p value
Intercept	−5.0337	0.4011	−12.549	<0.001
Boveq	0.6615	0.2645	2.501	<0.05
NDVIanu	0.5092	0.2831	1.799	NS
NDVIvaranu	−1.4185	0.4664	−3.041	<0.01

*NS, not significant at p<0.05; z, statistic.

## Discussion

Although some of the human cases of CCHF in Spain could be associated with farm animals, research on domestic species is limited (*38*). Most studies in Spain have focused on wild ungulates because of their relevance to CCHFV (*24*,*25*), but even so, farm animals and their ticks may pose a risk for humans through closer contact. By selecting small domestic ruminants, we aimed to identify the areas of greatest risk for transmission to persons in contact with them and thus complement the risk maps previously obtained by using red deer (*13*). Some of the CCHF patients in Spain were animal farmers (*38*). In the absence of an effective vaccine to protect humans against CCHFV infections, the only feasible measure to protect the population at risk is prevention of tick bites. If risk for infection is higher for an animal in a territory, it is also higher for a person in that territory because the virus is transmitted mainly by tick bites to animals and humans. The factors that predispose human contact with infected ticks or animals will determine the actual risk for CCHFV infection (*12*), although risk will be greater in areas more environmentally favorable for virus transmission. Our results provide public health authorities with information about which areas of Andalusia have the highest risk for CCHFV transmission for anyone linked to small ruminant production. That information will enable design of better infection surveillance programs in the region, optimizing the resources available for those programs and improving the cost:benefit ratio of preventive actions.

Our study is based on a representative subsample of small ruminants for the study region. However, we did not include other domestic species (cattle, equids) or wild ungulates, which are relevant for CCHFV and its vectors and could improve the predictive capabilities of the model. In the future, we recommend considering the full range of species involved in CCHFV transmission to improve the accuracy of risk maps. In this study we did not corroborate that antibodies were specific for CCHFV, which should preferably be done by comparative neutralization assays in Biosafety Level 4 facilities. However, numerous positive serum samples from wild ungulates in the ELISA used were confirmed as CCHFV positive by neutralization assays against different orthonairoviruses (I. García-Bocanegra, unpub. data). The manufacturer of the multispecies double-antigen ELISA test used confirms 98.9% sensitivity and 100% specificity after testing with a multitude of animal species (*29*).

In a previous study conducted in 2 areas of Andalusia (Córdoba and Cádiz Provinces), and using the IDVet double-antigen ELISA, CCHFV seroprevalence among domestic ruminants (including cattle, sheep, and goats) was 17.9% (*38*). The inclusion of cattle samples makes it difficult to compare reported seroprevalence with our findings because cattle host higher burdens of *Hyalomma* spp. ticks than do small domestic ruminants (*39*) and may thus be more prone to CCHFV exposure. That study also found higher seroprevalence than the seroprevalence that we report for areas of northern Spain, which are less favorable areas for *Hyalomma* spp. ticks (6.8%) (*38*). The higher antibody prevalence most likely results from including cattle in the survey. Our results also contrast markedly with the high antibody prevalence (76%–87%) observed in red deer in western Andalusia (*40*). Previous studies of small domestic ruminants from Africa, Asia, and Europe showed a wide range of seroprevalence, 0.4%–74% among sheep and 2.1%–66% among goats (*28*). Our results agree with those of some studies conducted in the Mediterranean region (*41*,*42*). The low individual seroprevalence observed for animals of both species indicates that sheep and goats are of less concern than cattle, horses, or wildlife for farmers and public health authorities.

Our selected model included horse/cattle density as a predictor of CCHFV exposure risk. The lack of association between wild ungulate abundance and CCHFV exposure risk most likely indicates a low rate of interaction with the small domestic ruminant farms selected for the study. The rate of interaction between wild ungulates and their ticks and small ruminants raised on extensive farms that are also used for large game hunting is probably higher, perhaps leading to a higher risk for exposure to CCHFV. Cattle and horses are relevant hosts for *H. marginatum* ticks (*6*,*23*,*32*,*41*) and may be more abundant than wild ungulates in the vicinity of small ruminant farms, so their association with CCHFV was not unexpected. Previous studies already described the relevant role of farm animals in the risk for exposure to CCHFV (*24*,*43*). Among domestic animals, global CCHFV seroprevalence is second highest among cattle, after camels (*44*). Thus, our findings suggest that a regional strategy should perhaps be implemented to better control ticks on farm animals. For sheep and goats, increasing the frequency of acaricide application may result in more effective tick control (*41*).

The best-fitted model also included 2 abiotic predictors, NDVI and annual NDVI variance, which would probably define the environmental (climatic) niche for CCHFV vectors in southern Spain. The distribution of ticks is limited not only by host distribution but also by a combination of host presence/abundance and environmental favorability (*17*,*45*). Ticks occupy only a subset of their host range because they undergo a large part of their cycle on the ground, where abiotic factors determine tick development and survival rates (*17*,*32*). One of the limiting factors for tick survival and activity is moisture level, a determinant of tick abundance and a relevant driver of CCHFV transmission risk (*13*,*17*). The negative relationship observed between annual NDVI variance and CCHFV exposure risk may suggest that areas with substantial fluctuations in vegetation productivity (e.g., seasonal croplands) are unfavorable for CCHFV vectors, a possibility that agrees with the low predicted spatial risk in the agricultural lands of the Guadalquivir River basin.

The spatial distribution of the farms with seropositive animals was heterogeneous; most were distributed south and east of the study region. Because our model showed that this distribution was associated with some biotic and abiotic factors of the farm neighborhood, we were able to capture the environmental niche for small domestic ruminant exposure risk to CCHFV. The model projection identified that the areas of Andalusia with the highest abundance of wild ungulates (mainly red deer, wild boar, and Iberian ibex [*Capra pyrenaica*]) (*46*) had the highest risk for exposure to CCHFV. As previously observed for red deer (*13*), the predictive model not only projects the risk for small domestic ruminants but also for other hosts of CCHFV vector ticks. The model identified the areas of highest risk to be Los Alcornocales Natural Park (Cádiz and Málaga Provinces), the area surrounding the Doñana National Park (south of Huelva Province), most of the Sierra Morena mountain chain, and the Sierras de Cazorla, Segura y Las Villas Natural Park (northeastern Jaén Province). Sánchez-Seco et al. (*26*) found CCHFV-positive *Hyalomma* spp. ticks in Los Alcornocales Natural Park, whereas we detected high CCHFV prevalence among ticks and high antibody levels in the wild ungulates of Doñana National Park (*24*) and identified Los Alcornocales Natural Park, Doñana National Park, and the Sierra Morena mountain chain as high-risk areas (*13*,*25*). Recently, we found that ≈30% of wild boar in Sierras de Cazorla, Segura y Las Villas Natural Park have antibodies against CCHFV (*47*). Our risk map identifies areas of low and high risk that were identified on larger spatial resolution on a map generated for Spain from a model based on red deer (*13*). However, the limited sensitivity (10.2%) of our model to predict the risk for exposure of small domestic ruminants to CCHFV prevents us from detecting all areas where CCHFV may be circulating among small domestic ruminants in Andalusia. Therefore, in the future, it would be desirable to base estimates of CCHFV actual distribution in Andalusia on vector population dynamics and CCHFV prevalence among the vectors. Comparison of the findings of the small domestic ruminant-based model with existing evidence on the prevalence of CCHFV infection/exposure and the predictive outcome of wildlife-based risk models indicates that despite its limited predictive sensitivity and tendency to false negatives, our model can capture spatial foci of high and low CCHFV risk. Consequently, despite the observed limitations, it may constitute a useful tool for preventing cases of CCHF in humans. The high specificity of the model indicates that the identified low-risk hotspots are actually zones with low risk for exposure. We conclude that modeling of CCHFV exposure risk for small domestic ruminants, although at low rates of virus exposure, is a useful tool for mapping CCHFV transmission risk hotspots and preventing CCHF in humans, at least in the study area.
